# MRI characteristics for “phantom” scratching in canine syringomyelia

**DOI:** 10.1186/s12917-017-1258-2

**Published:** 2017-11-16

**Authors:** Zoe R. Nalborczyk, Angus K. McFadyen, Jelena Jovanovik, Anna Tauro, Colin J. Driver, Noel Fitzpatrick, Susan P. Knower, Clare Rusbridge

**Affiliations:** 1 School of Veterinary Medicine, Faculty of Health & Medical Sciences, Daphne Jackson Road, Guildford, Surrey, GU2 7AL UK; 2 akm-stats, Glasgow, Scotland, UK; 3 Fitzpatrick Referrals Orthopaedics and Neurology, Halfway Lane, Eashing, Godalming, Surrey, UK

**Keywords:** Neuropathic itch, Alloknesis, Superficial dorsal horn, Fictive scratch, Central pattern generator, Chiari malformation, Chiari-like malformation

## Abstract

**Background:**

A classic sign of canine syringomyelia (SM) is scratching towards one shoulder. Using magnetic resonance imaging (MRI) we investigate the spinal cord lesion relating to this phenomenon which has characteristics similar to fictive scratch secondary to spinal cord transection.

Medical records were searched for Cavalier King Charles spaniels with a clinical and MRI diagnosis of symptomatic SM associated with Chiari-like malformation (CM). The cohort was divided into SM with phantom scratching (19 dogs) and SM but no phantom scratching (18 dogs). MRI files were anonymised, randomised and viewed in EFILM ™. For each transverse image, the maximum perpendicular dimensions of the syrinx in the dorsal spinal cord quadrants were determined. Visual assessment was made as to whether the syrinx extended to the superficial dorsal horn (SDH).

**Results:**

We showed that phantom scratching appears associated with a large dorsolateral syrinx that extends to the SDH in the C3-C6 spinal cord segments (corresponding to C2-C5 vertebrae). Estimated dorsal quadrant syrinx sizes based on the perpendicular diameters were between 2.5 and 9.5 times larger in dogs with phantom scratching, with the largest mean difference *p*-value being 0.009.

**Conclusion:**

SM associated phantom scratching appears associated with MRI findings of a large syrinx extending into the mid cervical SDH. We hypothesise that damage in this region might influence the lumbosacral scratching central pattern generator (CPG). If a scratching SM affected dog does not have a large dorsolateral cervical syrinx with SDH involvement then alternative explanations for scratching should be investigated.

**Electronic supplementary material:**

The online version of this article (10.1186/s12917-017-1258-2) contains supplementary material, which is available to authorized users.

## Background

**Fig. 1:**
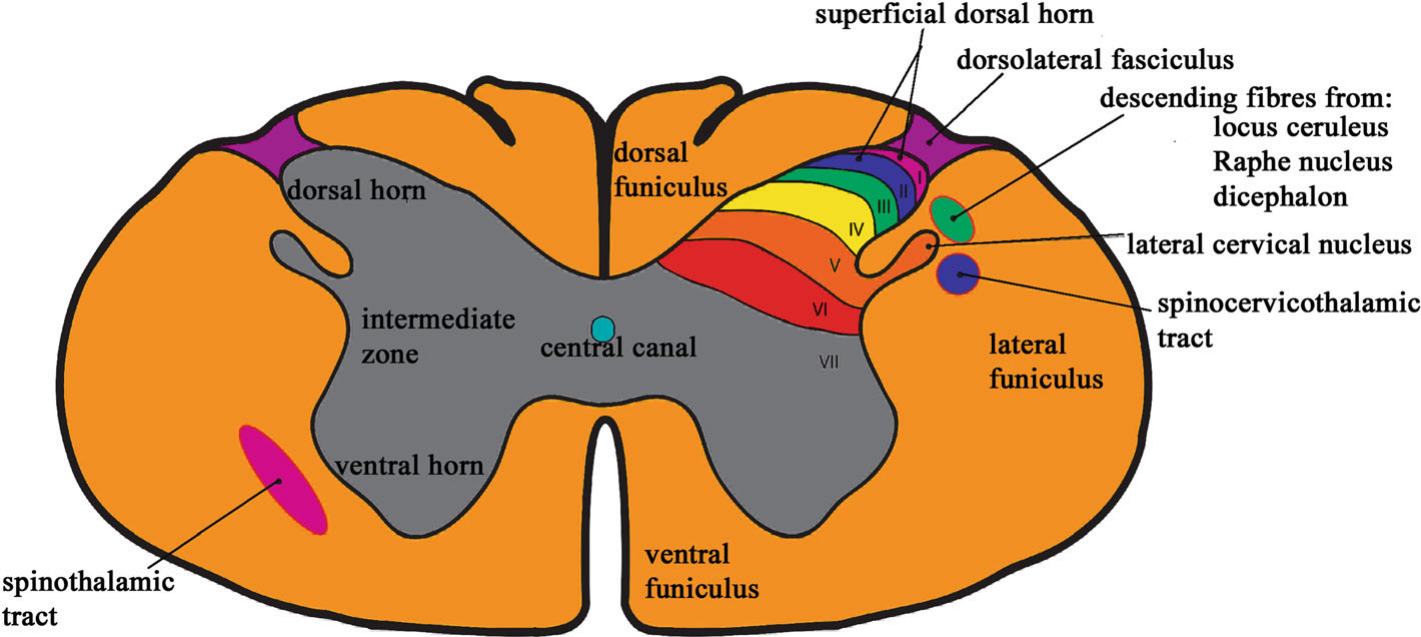
Pivotal spinal cord anatomy for nociception, puritoception and perception of pain and itch. Lamina of the spinal cord dorsal horn are indicated by roman numerals. The superficial dorsal horn is the main target for nociceptive and prurioceptive afferents whereas the deeper laminae receive light touch, proprioception and vibration afferents. The other neuronal constituents of the dorsal horn include local and descending axons which modulate sensory transmission. The projection neurons for nociception travel via the spinocervicothalamic (via the lateral cervical nucleus) and spinothalamic tract to the thalamus and then onto the brain’s pain processing centres that lead to conscious and unconscious pain and itch perceptions, and the emotions, and actions that these evoke. There is much overlap between neural circuitry of nociception, pruritoception, pain and prurititis because both are nocifensive and it is postulated that itch evolved from simple nociception to protect against small clinging threats [12]. However in carnivores the specific pathway for ascending pruritoceptive information in the carnivore has not been investigated. The brain modulates as well as receives information about nociception and itch and can inhibit or potentiate impulses. Scratching blocks the perception of itch [32]. Inhibition of the itch signal is mediated by dorsal horn inhibitory interneurons which are thought to be influenced by inhibitory noradrenergic and serotoninergic neurons descending from the diencephalon, raphe nuclei and midbrain periaqueductal grey mater (locus ceruleus) via the rostral ventral medulla [44]. These descending fibres are within the dorsolateral funiculus lateral to the dorsal horn


Syringomyelia (SM) is characterised by fluid filled cavities in the spinal cord. A classic sign of severe SM in the dog is a tendency to scratch towards one shoulder or neck region without skin contact, often referred to as “phantom scratching”. This sign has been associated with the disease since its first description [1]. The cavalier King Charles spaniel breed (CKCS) is predisposed and presence of SM correlates with a more extreme Chiari-like malformation (CM) [2]. CM is a complex developmental malformation of the skull and cranial cervical vertebrae occurring ubiquitously in the CKCS that is characterized by rostro-caudal bony insufficiency resulting in conformational change and overcrowding of the brain and cervical spinal cord particularly at the craniocervical junction. Traits that increase risk of SM in the CKCS include rostral displacement of the atlas and axis, acute angulation between the sphenoid and basioccipital bone, reduced occipital crest and increased cervical flexure and odontoid (dens) angulation [2, 3]. Although CM and SM can be asymptomatic: both disorders can be associated with a spatio-temporal gait change [4] and many dogs are presented with signs of pain - for example reluctance to exercise, vocalisation on lifting or sudden posture change and rubbing or scratching (with contact) at the back of the head or ears. [2, 5, 6]. SM can be associated with other neurological deficits including scoliosis, weakness and phantom scratching (without skin contact) [5–7]. SM associated phantom scratching can have a major impact on quality of life as leash walking can be difficult because touch from the neck collar or harness can induce the action. The action has paroxysmal and possibly involuntary quality which can also be triggered by excitement or movement (Additional file 1). Although simple to describe and recognise, the mechanism behind this action has been less easy to elucidate. A popular explanation is that affected dogs suffer neuropathic pain and/or itch and experience alloknesis (itch evoked by lightly touching the surrounding skin) or paraesthesia (a spontaneous or evoked sensation). Nevertheless the inadequately answered questions are: 1) if affected dogs experience unusual sensations, why do they make little or no skin contact? 2) If the explanation that the reason for lack of contact is that it is too painful (allodynia), why do some owners of dogs with severe phantom scratching state unequivocally that they do not believe their dog to be in pain and that it is the persistent action that compromises the dog’s quality of life? Scratching is a conscious, controlled motor response, targeted to the perceived site of itch and evolved as a defence against environmental threats such as clinging parasites [8, 9]. Similar to nociception, pruritoceptive information activates a specialised subpopulation of c-fibres which synapse in the superficial dorsal horn (laminae I and II) [10]. The spinal cord anatomy is represented in Fig. 1. Scratching removes the threat and diminishes the sensation of itch [9]. Like nociception, puritoception is a defence mechanism but chronic pruritus or pain is a debilitating disease. Neuropathic itch, implying intractable itch due to nervous system damage, is characterised by skin trauma and even mutilation [10–12]. Yet a hallmark of SM phantom scratching is the lack of purposeful skin contact and the description of SM phantom scratching is very similar to fictive scratch first described over one hundred years ago by Sherrington [13]. Sherrington showed that approximately three months after transection of the caudal cervical spinal cord in dogs, stimulation of the skin in the scapular region caudal to the transection level induced a rhythmic and non-purposeful scratching action of the ipsilateral limb scratching towards, but not making contact with, the skin. He further described that there was a curvature of the body and neck with the “head partially turned back for the foot more readily to reach it” [13]. The similarity of this description to SM phantom scratching can be appreciated in the accompanying video (Additional file 1). Like dogs with SM, fictive scratch is characterised by a receptive field where stimulation of the skin induces the scratching action. Sherrington hypothesised that there was a spinal central pattern generator (CPG) for scratching and that this had evolved as a protective response against clinging parasites and other irritants [13]. The similarity of phantom scratch to fictive scratch in a spinalised animal offers a hypothesis that phantom scratching may also be a consequence of a hyperactive pathway involving the lumbosacral scratching central pattern generator and that it is a phenomenon that is distinct from neuropathic pain i.e. dogs with phantom scratching may not have behavioural signs of pain and vice versa.

Additional file 1: Movie of phantom scratching” in a CKCS. There is a rhythmic and non-purposeful scratching action of the pelvic limb towards the shoulder usually not making contact with the skin. The trunk flexes and the head turns towards the scratching limb. This action can be elicited or exacerbated by excitement, exercise, touch and wearing of neck collars and harnesses. (Video courtesy of Ms. J Harrison, Passionate Productions). (MOV 97128 kb)


This investigation considered two possible hypotheses:

1. Phantom scratching is associated with a large dorsolateral syrinx in a specific cervical spinal cord segments. We were particularly interested in whether there was involvement of the first two cervical segments containing the lateral cervical nucleus and relay for ascending spinocervicothalamic tract fibres (Fig. 1). The spinocervicothalamic tract is the dominant pathway for nociception in carnivores in comparison to primates where the spinothalamic tract is more important [14, 15]. In primates prurioceptive information ascends the spinothalamic tract (contralateral lateral funiculus) to the midbrain periaqueductal grey matter, thalamus and higher centres [16, 17]. The specific pathway for ascending pruritoceptive information in the carnivore has not been investigated.
2. The phenomenon of phantom scratching is not just associated with a dorsolateral syrinx but one that extends to the superficial dorsal horn (SDH) (lamina I and II). The SDH (lamina 1/marginal zone and lamina 2/substantia gelatinosa) is the main target for nociceptive and prurioceptive afferents whereas the deeper laminae receive light touch, proprioception and vibration afferents (Fig. 1).

In addition, we hoped that this study could generate new hypotheses about neural pathways associated with scratching and fictive scratch that could be investigated in planned histopathological studies.

## Methods

### Cohort selection

Medical records from a 2 year period (March 2013–2015) at Fitzpatrick Referrals Orthopaedics and Neurology were searched for CKCS that had undergone MRI (119 dogs). The medical records were analysed by CR. Ten dogs were excluded because the imaging did not include the brain and cervical spinal cord, 1 dog was excluded because of a diagnosis of central nervous system inflammatory disease and 19 dogs were excluded because of incomplete medical records, diagnosis of skin disease and/or the cause of pain or scratching was equivocal, and/or because the dogs had not been examined by an ECVN diplomat/resident. This included 16 CKCS that were presented for health screening prior to breeding. The remaining 89 dogs were divided into phenotypical groups according to clinical and SM status and 49 dogs without clinical signs associated with SM were further excluded leaving two groups: 1) SM with phantom scratching (19 dogs) 2) dogs with clinical signs of SM including behavioural signs of pain but no phantom scratching (21 dogs). For the purposes of this study, SM was defined as a fluid filled cavity equal to or greater than 2 mm in diameter associated with CM detected on MRI. The presence of phantom scratching was defined as a repetitive scratching action towards the neck region which could be induced by stimulation of a receptive field in the cervical region and/or induced by excitement or exercise in the absence of generalised pruritus and when clinical examination did not suggest skin and external ear disease (for example skin reddening or crusting) and when the scratching behaviour had been observed by an ECVN diplomate or resident. After review of the MRI, three cases were subsequently excluded as their MRI did not include transverse imaging of the cervical spinal cord. The remaining total study cohort therefore comprised 19 dogs with phantom scratching (“scratchers”) and 18 dogs with SM but no phantom scratching (“non-scratchers”).

### MRI examination

All MRI examinations were performed on 1.5 T scanner (Symphony Maestro Class, Siemens, Enlargen, Germany) with dogs under general anaesthetic. All dogs were positioned head first in dorsal recumbency and a combination of head and spine coil was used for all studies. The studies included routine T2-weighted MRI sequences in sagittal and transverse plane of the cranium and spinal cord. Only images from the cervical spinal cord were included in the study. The transverse sequences were centred on the widest part of a syrinx and covered the entire cervical syrinx. The study parameters for the sagittal plane were TR 2700 ms, TE 104 ms, FOV 220 mm, Slice thickness 2 mm, Matrix 512 × 512, whereas parameters for the transverse plane were TR 3200 ms, TE 109 ms, FOV 130 mm, Slice thickness 3–3.5 mm, Matrix 384 × 384.

### Measurements

The MRI studies were anonymised and randomised by JJ who was blinded to the clinical status. The MRI studies were viewed by DICOM viewing software eFILM workstation (Merge Healthcare 900 Walnut Ridge Drive, Hartland, WI 53029 USA).

#### Investigation 1: Is phantom scratching associated with a large dorsolateral syrinx in a particular region of the cervical spinal cord i.e. certain cord segments?

**Fig. 2:**
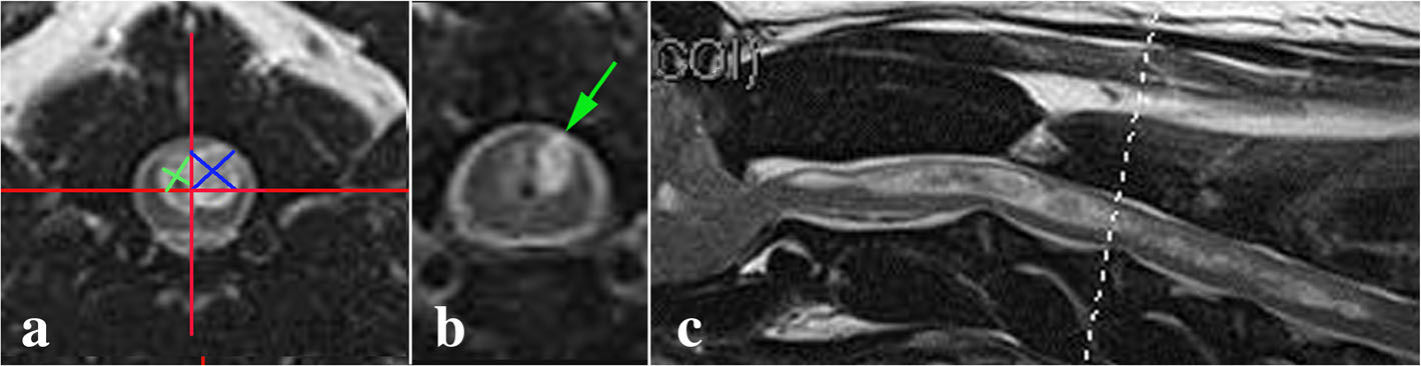
Study measurements and observations **a**: Transverse T2-weighted image of spinal cord at C3/C4. Using Efilm ™ the transverse spinal cord is divided into quadrants (red lines) by first placing a vertical line bisecting the spinous process and then placing the horizontal line to bisect the spinal cord and the vertical line at 90°. The perpendicular diameters of the syrinx in each dorsal quadrant are measured (blue lines – 0.4 × 0.4 cm; green lines 0.3 cm × 0.2 cm). **b**: Transverse T2-weighted image of spinal cord at C1. The green arrow illustrates a syrinx which extends to the area of the SDH. **c**: Midsagittal T2-weighted spinal cord with SM. White dotted line indicates the transverse section at C3/C4 for 3a

For each transverse MRI slice in cervical area, presence or absence of a syrinx was recorded. If present, the extent of the syrinx within the spinal cord was determined by measuring the maximum perpendicular dimensions of the cavity in each spinal cord dorsal quadrant in each transverse MRI section (Fig. 2). After measurements were completed the maximum dimensions of the syrinx (in cm) in each dorsal quadrant in each transverse MRI section were multiplied to give a value that reflected the perpendicular dimensional area of the cavity in each quadrant for each transverse MRI section. Subsequently a figure representing the dorsal syrinx cavity size for each spinal cord segment was obtained by calculating the mean of all the perpendicular dimension areas for each transverse MRI section for each segment for each dog.

#### Investigation 2: Is phantom scratching associated with a dorsolateral syrinx that extends to the SDH?

A visual assessment was made as to whether the syrinx extended to the SDH in each transverse MRI slice. This was defined as extension of the syrinx to the dorsolateral margin of the spinal cord so that that rim of spinal cord was not or only just appreciable and that the fluid filled cavity of the syrinx appeared confluent with the subarachnoid space (Fig. 2b). The position of each transverse slice relative to the vertebral body or intervertebral disc was noted (e.g. mid C2 vertebrae).

All measurements were made by ZN after an initial period of training. After this period of training and several hours of practice, 2 cases (each with 189 measurements for Investigation 1 and 30 assessments for Investigation 2) were determined independently by ZN and CR to determine inter-observer reliability. ZN then made an independent second measurement to determine intra-observer reliability.

Spinal cord segments were assumed as depicted in Table 1.

**Table 1:** Relationship between cervical spinal segments and vertebrae

Cervical Spinal cord segment	Vertebral and intervertebral disc (IVD) relationship
C1	Craniocervical junction
C2	C2 dens and C1
C3	C1/C2, C2 and C2/C3 IVD
C4	C3 and C3/C4 IVD
C5	C4 and C4/C5 IVD
C6	C5 and C5/C6 IVD
C7	C6
C8	C6/C7 IVD, C7

Relative position of cervical spinal segments and vertebrae in the dog [15]

### Statistics

Measurement reliability was assessed for inter and intra observer reliability using intra-class correlation [ICC] model [1, 2] [18] and the kappa statistic where appropriate. For Investigation 1 quadrant areas were summarised using mean, standard deviation, median along with minimum/maximum. These values were calculated from all the measurements from each transverse MRI section for each dog. Due to the non-Normality of the data, comparisons between scratchers and non-scratchers were made using Mann–Whitney tests at a 5% level of significance. In Investigation 2, cross-tabulations of Group by whether syrinx was extended to the SDH or not were constructed for each spinal segment and, where appropriate, chi-square tests were used to assess any associations. Fisher’s exact tests were then used to test differences in the proportion of dogs within each group with an extended syrinx.

## Results

The average age of the “scratchers” was 5.0 years (range 1.2–10.6 years) and 8 of 19 dogs were male. The average age of the “non-scratchers” was 6.9 years (range 2.6–13.7 years) and 11 of 18 dogs were male. Inter-rater reliability of syrinx identification based on the small sample of dogs was found to be good with significant kappa values in excess of 0.75 and the ICC results for the slice measurement within each quadrant were all significant and in excess of 0.8. Intra-rater reliability of syrinx identification was excellent with very significant kappa values of 1 and the ICC results for the slice measurement within each quadrant were all significant and in range 0.831 to 0.994 with only one value below 0.9.

### Investigation 1. Is phantom scratching associated with a large dorsolateral syrinx in a particular region of the cervical spinal cord i.e. certain cord segments?

Approximately 5000 measurements and observations were made and the full dataset is available (Additional file 2). Table 2 illustrates a summary of the findings.

**Table 2:** Summary statistics for mean syrinx perpendicular dimensions (cm^2^) in each dorsal quadrant

Spinal segment	Mean	SD	Median	Min Max	Two sample test (*p*=)	SAMPLE SIZE
C2 LQ						
SR	0.0248	0.0153	0.0256	0.0025/0.0450	0.121	6
NSR	0.0428	0.0194	0.0467	0.0217/0.0600		3
C2 RQ						
SR	0.0119	0.0136	0.0069	0.0025/0.0313	0.032	4
NSR	0.0425	0.0152	0.0350	0.0325/0.0600		3
C3 LQ						
SR	0.1020	0.0480	0.0944	0.0103/0.1789	**0.001**	15
NSR	0.0331	0.0290	0.0175	0.0050/0.0943		14
C3 RQ						
SR	0.1021	0.0236	0.1033	0.0550/0.1369	**<0.001**	15
NSR	0.0274	0.0220	0.0171	0.0063/0.0743		14
C4 LQ						
SR	0.1122	0.0571	0.1060	0.0200/0.2220	**<0.001**	19
NSR	0.0457	0.0420	0.0233	0.0090/0.1250		14
C4 RQ						
SR	0.1172	0.0601	0.1000	0.0222/0.2400	**<0.001**	19
NSR	0.0447	0.0400	0.0272	0.0085/0.1233		14
C5 LQ						
SR	0.1013	0.0607	0.0957	0.0063/0.2275	**<0.001**	15
NSR	0.0212	0.0218	0.0125	0.0050/0.0740		12
C5 RQ						
SR	0.0993	0.0501	0.0900	0.0242/0.1925	**<0.001**	15
NSR	0.0225	0.0230	0.0135	0.0030/0.0780		12
C6 LQ						
SR	0.0876	0.0535	0.0930	0.0200/0.1625	**0.002**	8
NSR	0.0089	0.0041	0.0100	0.0038/0.0146		5
C6 RQ						
SR	0.0653	0.0478	0.0500	0.0200/0.1300	**0.009**	8
NSR	0.0132	0.0088	0.0100	0.0050/0.0250		5
C7 LQ						
SR	0.1000	0.0652	0.1000	0.0100/0.1900	n/a	5
NSR	0.0133	–	0.0133	0.0133/0.0133		1
C7 RQ						
SR	0.0448	0.0249	0.0450	0.0100/0.0800	n/a	5
NSR	0.0200	–	0.0200	0.0200/0.0200		1
C8 LQ						
SR	0.3450	–	0.3450	0.3450/0.3450	n/a	1
NSR						0
C8 RQ						
SR	0.0600	–	0.0600	0.0600/0.0600	n/a	1
NSR						0

A figure representing the syrinx cavity area was obtained by multiplying the perpendicular dimensions of the cavity in each dorsal quadrant in each transverse MRI section. Subsequently a figure representing the dorsal syrinx cavity size for each spinal cord segment was obtained by calculating the mean of all the perpendicular dimension areas for each transverse MRI section for each segment for each dog. C – cervical (see Table 1) RQ – right dorsal quadrants LQ – left dorsal quadrants SR – scratcher, NSR – non-scratcher. C1 Not included due to sample sizes. Blank cell means that there was no syrinx at this site. Bold font - *p* values indicates a significant result (*p* < 0.05)

**Fig. 3:**
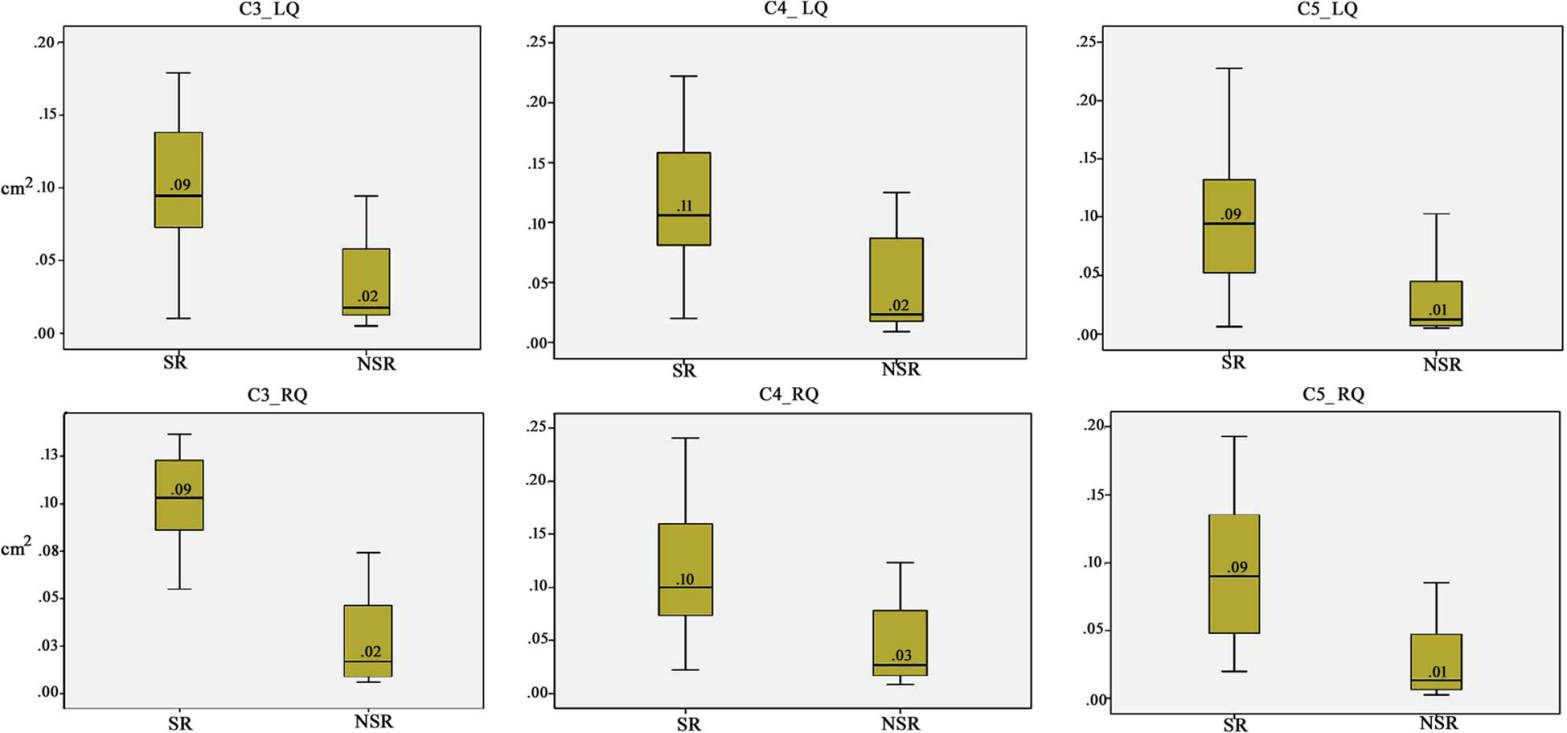
Boxplots representing the data from Table 2 for mean syrinx cavity diameters in each dorsal quadrant. Estimated dorsal quadrant syrinx cavity diameters (based on a multiplication of the perpendicular dimensions in each transverse MR slice) were between 2.5 and 9.5 times larger for “scratchers”, with the largest mean difference *p*-value being 0.009. Y axis scale in cm^2^

**Fig. 4:**
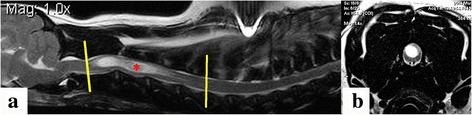
MRI from a CKCS with phantom scratching. **a** Midsagittal T2-weighted image of the cervical spinal cord. This study suggested that phantom scratching is associated with a large dorsolateral syrinx within the C3-C6 spinal segment (between the yellow lines). 100% of “scratchers” had a large dorsolateral syrinx within the C4 spinal segment (vertebrae C3; red asterisk). **b** Transverse T2-weighted image of the spinal cord at C3 illustrating a large dorsolateral syrinx that extends to the SDH

The study found that there was greater dorsal quadrant involvement in the scratcher group for spinal cord segments C3 – C6 (vertebrae C2 – C5). This suggested that phantom scratching may be associated with a large dorsolateral syrinx in the mid cervical spinal cord and illustrated by boxplots (Fig. 3). 100% of the 19 scratchers had large syringes in the dorsal quadrants of spinal segment C4 (vertebrae C3) indicating that this might be a particular region of interest in future studies. (Fig. 4). Spinal segments C1, C2, C7 and C8 had low numbers of dogs and therefore either could not be tested statistically or the results should be viewed with caution.

### Investigation 2. Is phantom scratching associated with a dorsolateral syrinx that extends to the SDH?

The summary statistics are illustrated in |Table 3.

**Table 3:** Summary statistics for dogs with a syrinx extending to the SDH

Spinal segment	Scratchers (19 dogs)			Non-Scratchers (18 dogs)			Chi-square [*p*-value]	Fisher’s Exact *p*-value
	% SDH SM	# SDH	# SM	% SDH SM	# SDH SM	# SM		
C1	0	0	0	0	0	0	n/a	n/a
C2	0	0	0	0	0	0	n/a	n/a
**C3**	**89.5**	**17**	**19**	**11.1**	**2**	**18**	**19.7 [<0.001]**	**<0.001**
**C4**	**83.3**	**15**	**18**	**14.3**	**2**	**14**	**12.4 [<0.001]**	**<0.001**
**C5**	**64.7**	**11**	**17**	**0**	**0**	**12**	**9.9 [0.002]**	**<0.001**
**C6**	**62.5**	**5**	**8**	**0**	**0**	**5**	**2.8 [0.095]**	**0.044**
C7	40	2	5	0	0	1		0.667
C8	0	0	0	0	0	0	n/a	n/a

% SDH SM – proportion of dogs that had a syrinx which extended to superficial dorsal horn

# SDH SM - number of dogs that had a syrinx which extended to superficial dorsal horn

#SM - number of dogs that had a syrinx at that spinal cord segment. Bold font - *p* values indicates a significant result (*p* < 0.05)

Scratchers were significantly more likely to have SDH involvement in the C3 to C5 spinal segments (C2 to C4 vertebrae) and less so for the C5 spinal segments (C6 vertebra). There was a marginal significant difference for the C6 spinal segment (C7 vertebrae) and no significant difference at C7. The number of dogs that had a syrinx extending to the SDH was low, once again, for spinal cord segments C6 and C7. Spinal cord segments C1 and C8 did not have a syrinx in any dog. In addition scratchers were more likely to have SDH involvement over several transverse slices. The mean ± standard deviation and median number of affected slices was respectively 10.8 ± 6.8 and 10 (range 0–28) for scratchers and 0.6 ± 1.7 and 0 (range 0–7) for non-scratchers.

## Discussion

### What is the neuroanatomical site pivotal for the phenomenon of phantom scratching?

This study suggested that phantom scratching in the dog may be associated with a large dorsolateral syrinx in the C3-C6 spinal segments (C2-C5 vertebrae). This correlated with the region of syrinx extension to the SDH (Fig. 4). An earlier hypothesis by this group was that the first two cervical segments may be pivotal for phantom scratch. However this study found low numbers of dogs with syringes at this site and the hypothesis was not proved. The first two cervical segments were of interest for several reasons. First, they contain the lateral cervical nucleus (relay for ascending spinocervicothalamic tract fibres). Second, a model of fictive scratch can be created in the decerebrate/spinal cat by application of tubocurarine to the dorsal surface of the cervical cord at C1 (and to a lesser extent C2) and then rubbing the pinna and the skin behind the ear [19, 20].

Scratchers were also likely to have SDH involvement over many adjacent transverse MRI slices and therefore cervical spinal cord segments. This may be important because it is suggested that lamina I of the SDH is a functionally interconnected layer with local circuit neurons connecting the dorsal grey matter of several spinal cord segments [21]. From this study it might be concluded that a dog with MRI evidence of a syrinx extending to the SDH is at risk for phantom scratching. Conversely, if the syrinx in a scratching dog with SM does not extend to the SDH and/or there is not extensive dorsolateral spinal cord involvement, then another explanation for scratching should be sought before a diagnosis of SM associated scratching is made. In the authors’ experience, presentation with a history of caudal head and/or ear scratching/rubbing is not unusual with brachycephalic toy breed dogs with suspected CM-pain [2, 5]. However the nature of the scratching is different in such cases. In the authors’ experience, dogs with suspected CM-pain may scratch to the sides of the head/ears but make skin contact and may vocalise during or shortly before scratching (with ear disease excluded as a cause of these signs). Since the phenomenon of SM phantom scratching has been publicised by social media, there can be a risk of misdiagnosis. Some dogs with SM are asymptomatic and there are many alternative explanations for scratching, especially if the pruritus is generalised or of multiple digits.

### Neuronal population and neurotransmitters within the C3-C6 SDH neuroanatomical site which could be damaged or modified by a syrinx

Activation of a population of gastrin-releasing peptide receptor–expressing interneurons in the SDH is key in itch transmission [22, 23]. These pruritoceptive circuits are regulated by GABAergic inhibitory dorsal horn interneurons [22, 24]. Glutamate release is pivotal for itch modulation [25] which may explain why (anecdotally) gabapentin and pregabalin suppress SM phantom scratching, drugs commonly prescribed for this purpose. Other paramount molecular signalling include substance P, calcitonin-gene related peptide and serotonin [9]. Substance P binds to the tachykinin neurokinin 1 receptor (NK- 1R), Recent studies have indicated that although gastrin-releasing peptide receptor–expressing neurons contribute to hyperknesis, it is the NK-1R expressing neurons located in the dorsomedial aspect of the SDH that are pivotal to alloknesis or ongoing itch [26]. These NK-1R expressing neurones give rise to ascending somatosensory projections [26] and represent a potential target to treat chronic itch [27]. A NK-1R antagonist, maropitant citrate, can reduce the size of lesions in C57BL/6 mice with idiopathic ulcerative dermatitis, a rodent model of chronic itch, which is characterised by lesions of the neck and shoulders and scratching behaviour induced by substance P [28]. Nonetheless, although NK-1P mediated itch seems a plausible theory for phantom scratching, there is the paradox that phantom scratching seems to be associated with damage to the SDH and if this were true then this neuronal population may be compromised. Also there is an argument that phantom scratching is not the same as neuropathic itch because there is no skin trauma. It is therefore more logical to hypothesise that phantom scratching may be due to damage to an inhibitory GABA and/or glycine neuron population. In the spinal cord dorsal horn, 30–40% of all neurons that regulate the transmission of information that is eventually perceived as touch, pain and itch are inhibitory [29]. These inhibitory neurons are found in all laminae except laminae IX and in rodents, GABA and glycine expressing interneurons account for appropriate a quarter of the neuronal population in the SDH [30, 31]. Recent studies have identified a pivotal dorsal horn neuronal population which express neuropeptide Y and inhibitory interneuron markers [32, 33]. Loss of these neurons produces disinhibition of a presumed and unknown excitatory neuron. Importantly, this neuronal population is activated by mechanical stimulation and is independent from puritoceptor and histamine transmission [32, 33] i.e. represent a possible candidate cell for phantom scratching which is also triggered by mechanical stimulation. The next stage in this study is compare neuronal populations in histological sections from CKCS with syringomyelia in order to elucidate this hypothesis further.

### Can we better understand the mechanism of scratch and the scratching central pattern generators (CPG)?

Scratching is a stereotyped sequence of muscle contractions that may be produced in a reliable but flexible manner. These, and other regular movements such as locomotion, are controlled by local neural circuits or CPG that are capable of producing repetitive movements, even when isolated from the brain and sensory inputs [13, 34, 35]. Experiments have suggested that the CPG for pelvic limb scratching is in the lumbosacral segments [36]. Therefore, if phantom scratching was similar to fictive scratch, it would suggest that the syrinx damages a neuronal population which influences the descending projections to the lumbosacral CPG for scratching. It is hypothesised that a logical explanation could be loss of an inhibitory interneuron population and this results in a disinhibition of scratching neural circuits and (presumed) synaptic reorganisation. The link between the cervical dorsal horn and lumbosacral intumescence in fictive scratching is the propriospinal system [37]. Propriospinal neurons are interneurons which connect multiple spinal cord segments and participate in complex or “long” motor reflexes including modulating CPGs [38, 39]. They have considerable plasticity and are pivotal in functional recovery after incomplete spinal cord injury [39]. Long propriospinal neurons have cell bodies in the cervical dorsal horn and the axons course caudally both in the superficial layers of white matter in the ventral and the lateral funiculi to terminate in the lumbosacral grey matter [39]. Intriguingly spinal segments C3 and C4 are the site of a population of propriospinal neurons termed the C3-C4 propriospinal system. This is pivotal for control of forelimb voluntary movement which connect to propriospinal neurons that project to more caudal spinal segments [39, 40]. Given the site, it is tempting to consider this might be involved in SM phantom scratching however this is conjecture. Finally the role of C-bouton synapse (spinal cholinergic interneurons) in fictive locomotion (spinal walking) has recently been demonstrated [41]. These neurons original from interneurons located lateral to the central canal and are responsible for increased motor neuron excitability in fictive locomotion [41, 42]. As a potential natural model of fictive scratch, dogs with SM associated phantom scratching may represent a huge resource for understanding neural plasticity and further study is warranted.

### Study limitations

This study had limitations of which the reader should take account. The number of study animals was small. Furthermore our MRI protocol dictates that a block of transverse images are planned to cover the syrinx so that there is a “slice” perpendicular to the spinal cord through the widest part of the syrinx. This method ensures that the extent of the syrinx can be appreciated but meant that the exact position of the transverse slices varied between dogs. It also meant that syrinx free portions of the spinal cord were less likely to have transverse images. Every effort was made to compare like with like in terms of slices but perfect matching was inevitably not possible however the differences were considered to be occurring at random and the same frequency in both groups. Consequently measurements of the length of syrinx grey column involvement would have been inaccurate and therefore were not obtained. A further limitation was that measurements of thoracic and lumbar syringes were not included because of the inconsistency of area and completeness of the transverse imaging caudal to the cervical region. Therefore the possibility of more caudal influences on phantom scratching were not investigated and it should be considered that the association of a wide mid-cervical syrinx and phantom scratching may be a coincidence especially as the mid cervical region is the most common site for syrinx development [43]. A further limitation of the study was the definition of SDH involvement as extension of the syrinx to the dorsolateral margin of the spinal cord so that that rim of spinal cord was not or only just appreciable could be regarded as subjective and inaccurate. The “rule” that was followed during the study was that there was SDH involvement if the fluid in the syrinx and the subarachnoid space looked confluent. The smallest unit of measurement of eFILM is 1 mm so a more objective measure was not possible.

## Conclusions

This study suggested that phantom scratching in the dog is associated with a MRI appearance of large syrinx that extended to the SDH in the C3-C6 spinal segments (C2-C5 vertebrae). The results suggest that phantom scratching may occur after damage to mid-cervical SDH neurons. It is possible that this damage might influence activity of the lumbosacral scratching CPG. This hypothesis is now being investigated in histopathological studies.

## Additional files

Additional file 2: Dataset of syrinx measurements and observations. (XLSX 96 kb)

## Abbreviations

C: cervical (in reference to number spinal segment)
CKCS: Cavalier King Charles spaniel
CM: chiari-like malformation
CPG: central pattern generator
DICOM: Digital Imaging and Communications in Medicine
ECVN: European College of Veterinary Neurology
IVD: intervertebral disc
LQ: left dorsal quadrants
MRI: magnetic resonance imaging
NK- 1R: tachykinin neurokinin 1 receptor
NSR: non-scratcher
RQ: right dorsal quadrants
SDH: superficial dorsal horn
SM: syringomyelia
SR: scratcher
